# Undetermined Ruptured Low‐Grade Appendiceal Mucinous Neoplasm Following High‐Energy Blunt Abdominal Trauma Requiring Emergency Laparotomy

**DOI:** 10.1002/ccr3.70071

**Published:** 2025-01-02

**Authors:** Ippei Matsuo, Tetsuya Yumoto, Akari Tsuji, Ryo Tanabe, Toshihisa Matsumura, Mikoto Shimabara, Masaaki Akai, Shoji Takagi, Hiromichi Naito, Atsunori Nakao

**Affiliations:** ^1^ Department of Emergency, Critical Care, and Disaster Medicine, Faculty of Medicine, Dentistry, and Pharmaceutical Sciences Okayama University Okayama Japan; ^2^ Center for Graduate Medical Education Okayama University Hospital Okayama Japan; ^3^ Department of Digestive Surgery Japanese Red Cross Okayama Hospital Okayama Japan

**Keywords:** abdominal injuries, appendiceal neoplasms, computed tomography, mucinous, pseudomyxoma peritonei

## Abstract

Blunt abdominal trauma causing intraperitoneal injury and/or bleeding can be life‐threatening, requiring immediate intervention. Diagnosing these cases can be challenging, especially when pre‐existing conditions are involved. Low‐grade appendiceal mucinous neoplasm (LAMN) is a rare tumor of the appendix that can lead to pseudomyxoma peritonei. Herein, we present a case of ruptured LAMN following blunt abdominal trauma after a high‐energy head‐on collision, complicating the differentiation from other intraperitoneal injuries. A 42‐year‐old Japanese female was brought to our hospital following high‐energy head‐on collision. She presented with stable vital signs, complaining of anterior chest pain and abdominal tenderness without peritoneal irritation. Computed tomography scans indicated multiple fractures in her chest and complex fluid around the Douglas fossa extending to the ileocecal area, with a slightly dilated appendix tip. Despite stable vitals, emergency laparotomy was needed for suspected peritonitis and/or intraperitoneal hemorrhage. Emergency laparotomy revealed yellowish, jelly‐like ascites and a ruptured appendiceal tumor. LAMN was suspected, and the appendix was completely resected, with cytoreductive surgery carefully performed. Histopathological examination confirmed the diagnosis of LAMN. Postoperative course was uneventful, and the patient was discharged on Day 13 and referred for further LAMN management. This case report highlights the diagnostic difficulties of LAMN rupture following blunt abdominal trauma, stressing the need to consider rare conditions like LAMN in differential diagnoses of acute abdomen posttrauma.

**Summary**

- This case highlights the diagnostic challenge of distinguishing rare conditions, like low‐grade appendiceal mucinous neoplasm, in acute abdomen cases following trauma.
- Prompt identification and intervention are crucial to prevent complications, emphasizing the need to consider atypical neoplasms in trauma‐related differential diagnoses.

## 1 Introduction

Blunt abdominal trauma accompanied by intra‐abdominal bleeding and/or contamination is a significant cause of mortality and morbidity, often requiring prompt intervention. However, it can occasionally present a substantial diagnostic challenge [1]. While computed tomography (CT) is a standard tool for identifying major intra‐abdominal injuries, including bowel/mesenteric injury, initial evaluations can be difficult because findings like intraperitoneal fluid and mesenteric infiltration are often nonspecific, and free extraluminal air, a more definitive sign of injury, may not always be visible in the early stages [2].

Low‐grade appendiceal mucinous neoplasm (LAMN) is a rare tumor of the appendix that produces large amounts of mucin or gelatinous ascites, potentially leading to fatal consequences [3]. Patients are typically asymptomatic or exhibit nonspecific symptoms unless the tumor ruptures, which can result in an acute abdomen [4].

Herein, we present a case of initially undetermined ruptured LAMN following blunt abdominal trauma after a high‐energy head‐on collision, complicating the differentiation from other intraperitoneal injuries.

## 2 Case History/Examination

A 42‐year‐old Japanese female with a history of iron deficiency anemia was brought to our hospital 36 min after high‐energy trauma from a head‐on collision while wearing a seatbelt with a deployed airbag. Upon arrival at the emergency department, the patient was alert with the following vital signs: respiratory rate, 30 breaths/min; heart rate, 74 beats/min; blood pressure, 148/64 mmHg; body temperature, 36.2°C; and oxygen saturation: 99% on 10 L/min via a reservoir mask. The patient complained of anterior chest pain, had a laceration on her left lower thigh, and a contusion on the right groin. She exhibited mild to moderate tenderness in the right lower abdomen without signs of peritoneal irritation, such as guarding or Blumberg's sign.

## 3 Differential Diagnosis

Ultrasonography revealed a fluid effusion around the Douglas fossa. Laboratory findings showed elevated white blood cell count of 14,030 cells/mm^3^ (normal range: 3300–8600 cells/mm^3^), with segmented neutrophils at 68%, and lymphocytes at 22%. Hemoglobin level was 7.8 g/dL (normal range: 11.6–14.8 g/dL), lactate levels were normal at 1.8 mmol/L (normal range: < 2.0 mmol/L), and coagulation profile was normal.

**FIGURE 1:**
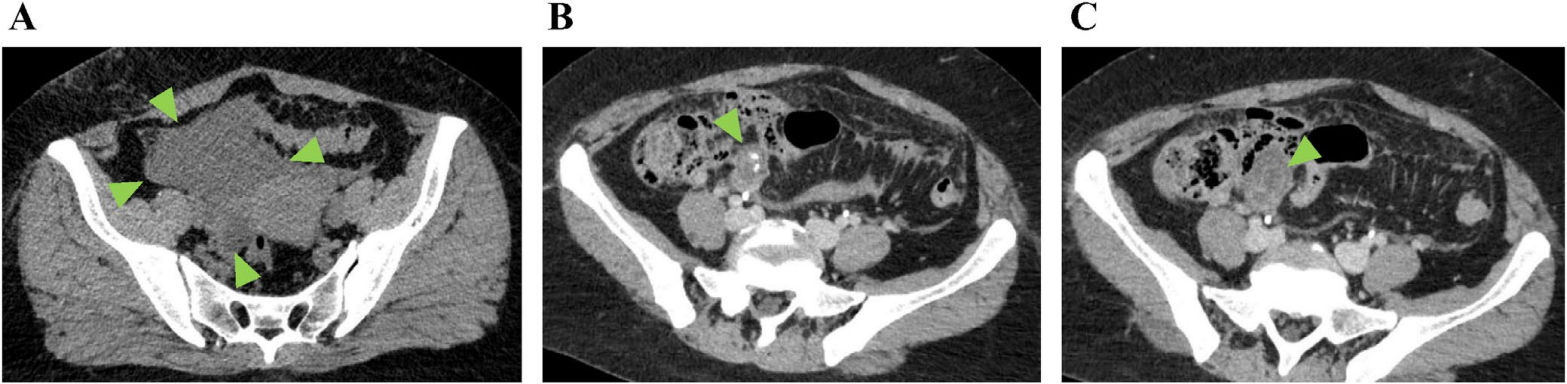
Abdominal computed tomography at presentation. (A) Plane image showing a peritoneal fluid around the Douglas fossa extending to the ileocecal area with a mean Hounsfield units of 28 (triangle arrow), indicating complex fluid due to intra‐abdominal injury. (B) Contrast‐enhanced image highlighting the extended tip of the appendix with a coprolith (triangle arrow). (C) Contrast‐enhanced image demonstrating an unclear appendiceal wall structure at the tip of the appendix (triangle arrow).

**FIGURE 2:**
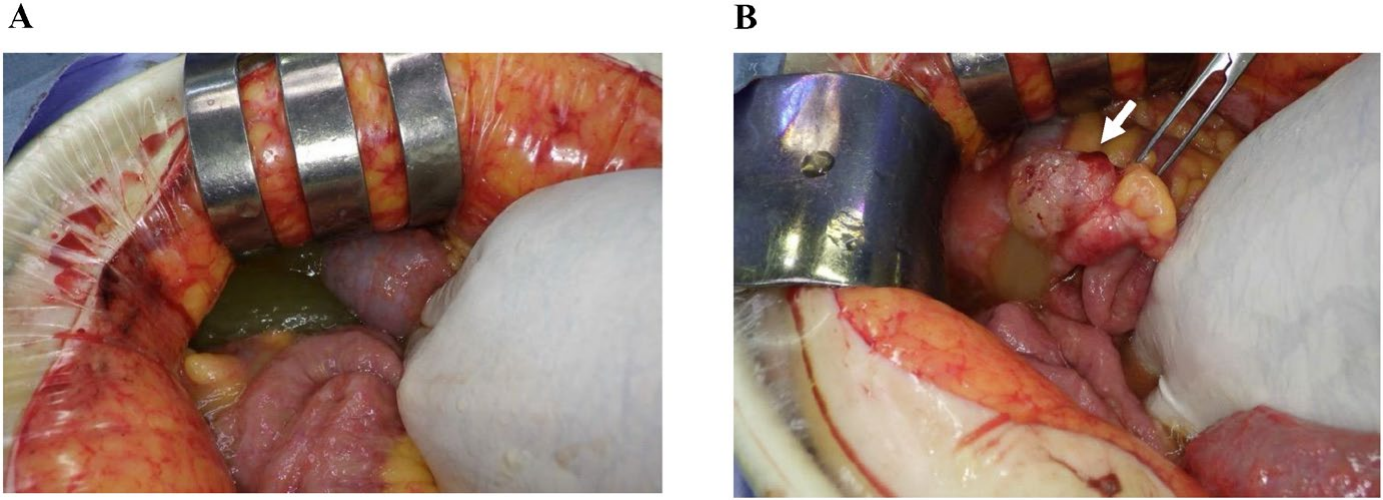
Intraoperative findings of emergency laparotomy. (A) Yellowish, clear, jelly‐like ascites were observed. (B) A ruptured appendiceal tumor extending from the mid to the tip of the appendix was found (arrow).

**FIGURE 3:**
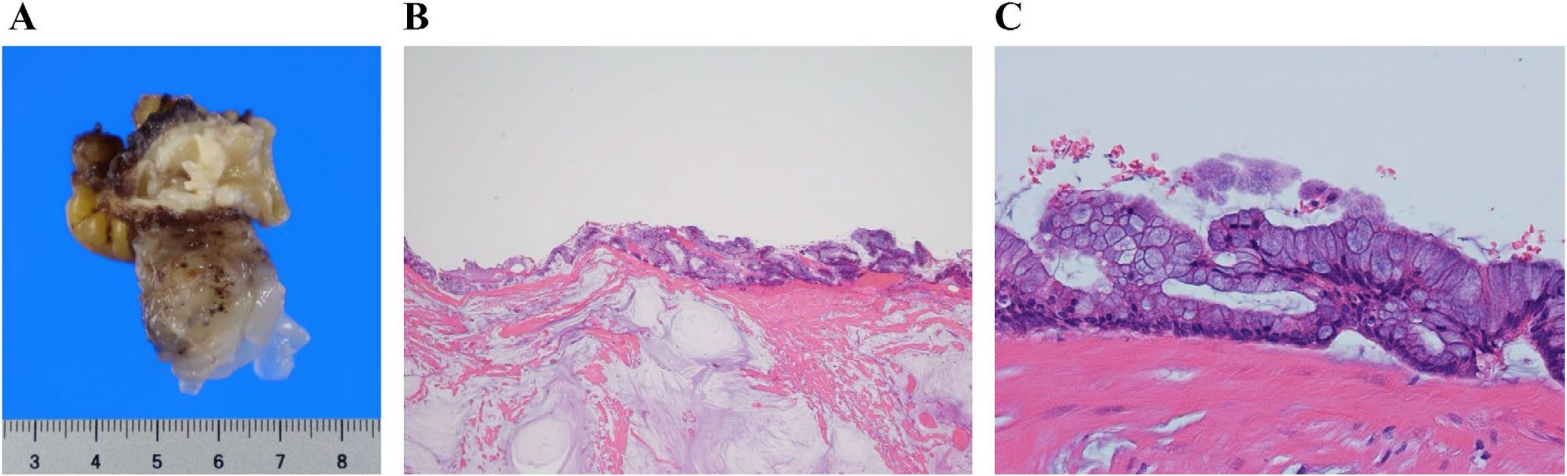
Histopathological findings of the resected tumor. A tumor was observed penetrating from the lumen of the appendix through the visceral peritoneum, with mucus exposed outside the appendix (A). The tumor is characterized by the villous proliferation of mucus‐producing tall columnar atypical epithelial cells, with mild to moderate nuclear atypia (B: H&E, 40×, C: H&E, 400×). No tumor invasion was observed in the mucosa of the appendiceal base.

CT of her torso revealed a right clavicle fracture, sternal fracture, 6th to 8th rib fractures on her left, and complex fluid around the Douglas fossa extending to the ileocecal area (Figure 1A). Subsequent contrast‐enhanced CT revealed a slightly extended tip of the appendix with a coprolith (Figure 1B) and an unclear appendiceal wall structure at the tip of the appendix (Figure 1C), without pneumoperitoneum or liver/spleen rupture. Although her vital signs were stable and hemoglobin levels remained at 7.5 g/dL with no decrease after an hour, emergency laparotomy was indicated due to localized peritonitis and a degree of intraperitoneal hemorrhage, possibly caused by the rupture of the appendix and/or mesenteric injury. Serial CT scans taken an hour apart showed an increase in peritoneal fluid extending from the appendix area through the lesser pelvis. Since the operation room was not available at our facility, the patient was then transferred to the closest tertiary hospital. Emergency laparotomy 5 h postinjury revealed a moderate amount of yellowish, clear, jelly‐like ascites extending from the right lower quadrant of the abdomen through the lesser pelvis, as well as a ruptured appendiceal tumor. There was no obvious intraabdominal bleeding or diffuse peritonitis (Figure 2A,B). Since a disseminated rupture of an appendiceal mucinous tumor was highly suspected, the procedure prioritized complete resection of the appendix to prevent further dissemination of mucinous material. The appendix was carefully mobilized to expose its base and resected to avoid rupture or spillage. The abdominal cavity and surgical wounds were then meticulously irrigated. Histopathological examination confirmed LAMN (Figure 3A: gross findings, Figure 3B: low power field, Figure 3C: high power field), classified as pT4a due to tumor rupture with mucin exposure on the serosal surface. Margins were evaluated, with no tumor cells found at the proximal margin of the appendix. The mucin present in the abdominal cavity was predominantly acellular; however, focal areas contained epithelial cells, suggesting a possible M1b designation.

The postoperative course was unremarkable. However, due to additional injuries requiring surgical fixation of her right clavicle fracture on Day 9, the patient was discharged on Day 13. Our surgical approach achieved an R0 resection; however, given the risk of pseudomyxoma peritonei, careful follow‐up was scheduled. The patient was referred to a specialized center for further evaluation and management of LAMN. At 6 months postsurgery, there were no obvious signs of recurrence. Follow‐up every 6 months is planned to monitor for recurrence, with consideration of cytoreductive surgery and hyperthermic intraperitoneal chemoperfusion if necessary.

## 4 Conclusion and Results (Outcome and Follow‐Up)

Due to pseudomyxoma peritonei risk, careful follow‐up was arranged. The patient was referred to a specialized center, and at 6 months postsurgery, no recurrence was observed. Our case report presents a unique instance of LAMN rupture induced by blunt abdominal trauma from a high‐energy head‐on collision, underscoring the diagnostic challenges in such scenarios. This case emphasizes the necessity of considering rare conditions like LAMN in the differential diagnosis of acute abdomen following trauma, especially given the potential for such tumors to mimic more common injuries and conditions.

## 5 Discussion

This report presents the first documented case of a LAMN rupture induced by blunt abdominal trauma from a head‐on collision accident, underscoring the diagnostic challenges associated with identifying intraperitoneal organ injuries in such cases.

LAMN is rare, with an incidence of 0.7%–1.7% among appendectomy specimens [5]. The clinical manifestation of LAMN varies widely, ranging from asymptomatic cases discovered incidentally during routine ultrasound examinations to presenting with right lower quadrant pain mimicking acute appendicitis or acute abdomen due to mucocele rupture [6]. Although larger LAMNs are considered more prone to rupture, the specific risk factors are not well defined. Since ruptures can occur during both open and laparoscopic surgery [7], blunt abdominal trauma is also believed to potentially trigger the rupture or exacerbate the perforation of these tumors. We assume that the rupture likely occurred earlier, with the blunt trauma exacerbating the perforation, as mucin is secreted by epithelial cells into the abdominal cavity in pseudomyxoma peritonei at an estimated growth rate of about 240 μm/h in humans [8], contributing to an increase in ascites over a short time interval following trauma. A variety of case reports of abdominal tumors rupturing secondary to blunt abdominal trauma have been published, including those involving the liver, pancreas, and uterus [9, 10, 11]. Additionally, a case of abdominal pain after abdominal trauma during boxing training, which led to the diagnosis of an unruptured but massive LAMN, has been reported [12]. However, the rupture of an appendix tumor, such as a LAMN in this case, is particularly noteworthy in this setting. Our case report highlights the importance of considering pre‐existing pathologies in the assessment and management of patients with blunt abdominal trauma, especially when peritoneal organ injury or hemorrhage is suspected, despite the condition's rarity.

Physicians should be familiar with CT findings of LAMN or its rupture, as it can sometimes mimic appendicitis, its rupture, or other conditions such as intraperitoneal injury following abdominal trauma. The slightly dilated appendix and presence of surrounding fluid seen in this case are often indicative of acute appendicitis, making it a key differential diagnosis on initial imaging. However, LAMN is typically characterized by distal luminal dilatation, with or without mural calcification, and a segment of morphologically normal appendix proximally [6, 13]. An appendiceal diameter greater than 15 mm is a useful indicator of chronic appendiceal obstruction, suggesting the possibility of an underlying appendiceal or cecal neoplasm. Additionally, reported CT values of intratumoral cystic contents via unenhanced CT range from 15 to Hounsfield units (mean, 20) [6], which is slightly lower than intraperitoneal hemorrhage, typically exceeding 30 Hounsfield units [14]. In the case of rupture, besides these features, a lobulated mucocele adjacent to the cecum would be a significant clue, highly suggestive of LAMN rupture [15]. It was extremely difficult to precisely diagnose LAMN preoperatively with only slight dilatation of the appendix, the CT value being very close to that of hemorrhagic ascites, and the absence of a clear “lobulated” mucocele, especially in the setting of treating high‐energy blunt abdominal trauma.

Given that the rupture of LAMN can lead to the release of cellular or acellular mucin, potentially causing pseudomyxoma peritonei, it is crucial to handle and resect the lesion with utmost care during surgery, to prevent further complications [16]. If the rupture is contained, a cecectomy or a right hemicolectomy may be considered; however, more extensive cytoreductive surgery should be performed only by experienced surgeons and only after reviewing the final pathology results [17]. Additionally, the abdomen and surgical wounds should be carefully irrigated to minimize the risk of tumor cell implantation. Patients with ruptured LAMN who are at risk for pseudomyxoma peritonei should be referred to a specialized center and monitored every 6 months to evaluate the need for potential cytoreductive surgery and hyperthermic intraperitoneal chemoperfusion [18].

We suspected organ injury due to trauma based on the accumulation of fluid in the abdominal cavity and opted for emergency surgery. Although the contrast‐enhanced CT did not show any extravasation of the contrast agent and the vital signs were stable, laparoscopic surgery was also considered to observe the abdominal cavity first. However, the high‐energy nature of the trauma and the associated risks ultimately led us to choose laparotomy. In cases of multiple trauma, particularly with suspected intra‐abdominal organ damage, such as bowel injury, laparotomy provides a more direct and comprehensive approach, allowing rapid control of potential bleeding and a thorough exploration of the abdominal cavity [19]. While laparoscopy is minimally invasive, it can be more challenging to assess and manage complex injuries swiftly in high‐energy trauma situations. Thus, laparotomy was chosen to ensure the patient's safety and stability, prioritizing effective and immediate intervention for any life‐threatening conditions. Despite the potential to avoid an invasive procedure, the urgency and complexity of the trauma guided this approach.

Our case highlights the diagnostic complexity of rare tumors like LAMN. Broader challenges remain in effectively screening neoplasms, including gynecologic cancers; while cervical cancer benefits from human papillomavirus vaccination, endometrial and ovarian cancers lack reliable screening, often leading to late diagnoses [20]. Emerging biomarkers are essential to advance early detection and improve outcomes across malignancies.

## Author Contributions

**Ippei Matsuo:** conceptualization, investigation, methodology, resources, visualization, writing – original draft. **Tetsuya Yumoto:** conceptualization, investigation, resources, visualization, writing – original draft. **Akari Tsuji:** conceptualization, investigation, methodology, visualization, writing – original draft. **Ryo Tanabe:** conceptualization, investigation, methodology, visualization, writing – review and editing. **Toshihisa Matsumura:** conceptualization, investigation, methodology, visualization, writing – review and editing. **Mikoto Shimabara:** conceptualization, investigation, methodology, visualization, writing – review and editing. **Masaaki Akai:** conceptualization, investigation, methodology, visualization, writing – review and editing. **Shoji Takagi:** conceptualization, investigation, methodology, visualization, writing – review and editing. **Hiromichi Naito:** conceptualization, resources, supervision, validation, writing – review and editing. **Atsunori Nakao:** conceptualization, resources, supervision, validation, writing – review and editing.

## Ethics Statement

Our institution does not require ethical approval when reporting individual case. This case report does not contain any research involving human or animals. Written informed consent has been obtained from the patient. The name of our institution is Okayama University Hospital Ethics Committee.

## Consent

Written informed consent has been obtained from the patient.

## Conflicts of Interest

The authors declare no conflicts of interest.

## Data Availability

The data from this case are available from the corresponding author upon reasonable request.
